# *Cryptosporidium muris*, a Rodent Pathogen, Recovered from a Human in Perú

**DOI:** 10.3201/eid0909.030047

**Published:** 2003-09

**Authors:** Carol J. Palmer, Lihua Xiao, Angélica Terashima, Humberto Guerra, Eduardo Gotuzzo, Gustavo Saldías, J. Alfredo Bonilla, Ling Zhou, Alan Lindquist, Steve J. Upton

**Affiliations:** * University of Florida, Gainesville, Florida, USA; † Centers for Disease Control and Prevention, Atlanta, Georgia, USA; ‡ Universidad Peruana Cayetano Heredia, Lima, Perú; § London School of Tropical Medicine, London, United Kingdom; ¶ United States Environmental Protection Agency, Cincinnati, Ohio, USA; # Kansas State University, Manhattan, Kansas, USA

**Keywords:** *Cryptosporidium muris*, Perú, zoonotic diseases

## Abstract

*Cryptosporidium muris*, predominantly a rodent species of *Cryptosporidium*, is not normally considered a human pathogen. Recently, isolated human infections have been reported from Indonesia, Thailand, France, and Kenya. We report the first case of *C. muris* in a human in the Western Hemisphere. This species may be an emerging zoonotic pathogen capable of infecting humans.

Cryptosporidiosis can be a debilitating diarrheal disease. While infections are normally acute and self-limiting in immunocompetent persons, cryptosporidiosis can be life threatening in those with compromised immune systems. In humans, cryptosporidiosis is caused predominantly by *Cryptosporidium parvum* or *C. hominis* (the latter was previously known as the *C. parvum* human genotype), and major outbreaks of the disease have been clearly associated with contaminated drinking water (*1*).

Recently, another species of *Cryptosporidium*, *C. muris*, has been suggested to be of concern to human health. *C. muris* is a parasite first identified in the gastric glands of mice (*2*). Experimental transmission studies have shown that the parasite readily infects multiple nonrodent hosts including dogs, rabbits, lambs, and cats (3). *C. muris*–like organisms have also been reported as opportunistic infectious agents in immunocompromised nonhuman primates (4). In the past 2 years, five cases of infections with *C. muris* or *C. muris*–like parasites have been reported from HIV-positive and healthy persons in Kenya (*5*), France (*6*), Thailand (*7*), and Indonesia (*8*). In this paper, we report on the first documented case of *C. muris* in a human in the Western Hemisphere. The parasite was recovered during the summer of 2002 in stools of an HIV-positive Peruvian woman with severe diarrhea. This finding was confirmed by light microscopy, polymerase chain reaction (PCR)–restriction fragment length polymorphism (RFLP), and DNA sequencing.

## The Study

In 2002, we conducted a year-long collaborative study on the epidemiology of *Cyclospora cayetanensis* infections in Perú. As part of that study, we collected approximately 100 stool samples in 2.5% potassium dichromate solution from persons in Lima and Iquitos with *Cyclospora* infection. Fecal samples were initially identified as *Cyclospora*-positive in Lima, and then transported to the United States for additional confirmation using wet mount and Nomarski interference contrast microscopy.

**Figure 1:**
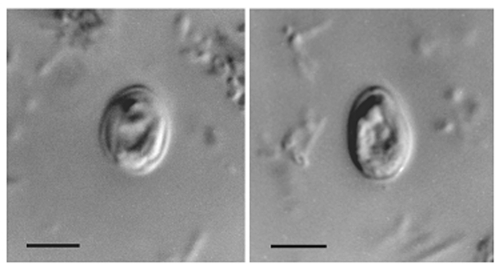
Nomarski interference contrast photomicrographs of *Cryptosporidium muris* from the feces of an HIV-positive human. Scale bars = 5 μm.

Two stool samples, which were taken on two sequential days from an HIV-positive woman who was 31 years of age, contained oocysts that appeared, based on morphology, to be *Cryptosporidium muris*. Low numbers of *Cyclospora cayetanensis* and *Blastocystis hominis* oocysts were also identified in the stool samples. The *Cryptosporidium muris* infection was initially identified by using wet mount microscopy with oocysts (n=25) averaging 6.1 (± 0.3) x 8.4 (± 0.3) μM (range 5.6–6.4 x 8.0–9.0) and a shape index (length/width) 1.38 (1.25–1.61) (Figure 1). Numbers of oocysts were determined semiquantitatively in each sample by hemacytometer, with an estimated 737,000 and 510,000 oocysts/g recovered from the submitted samples on day 1 and day 2, respectively. The diagnosis of *C. muris* was later confirmed through DNA analysis.

HIV was first diagnosed in the patient in November 2000 by using enzyme-linked immunosorbent assay and Western blot (immunoblot). She arrived at the hospital clinic in June 2002 with fever and reported that she had been experiencing diarrhea for >3 months. The patient reported that she had lost approximately 25 lbs. in the past 7 months, consistent with HIV-wasting syndrome. Her chest x-ray was abnormal, but four direct sputum examinations for acid-fast bacteria using Ziehl-Neelsen staining were negative, as were efforts at culturing *Mycobacterium tuberculosis*.

Other laboratory values for this patient at the time of stool sample collection were as follows: CD4 cell count 66/μL; hematocrit 36%; leukocytes 4,100/μL with 4% bands, 55% neutrophils, 27% lymphocytes, and 0% eosinophils; urine examination normal; creatine 0.8 mg/dL; urea 21 mg/dL; glucose 105 mg/dL; serum glutamic oxalacetic transaminase 30 IU/L; serum glutamic pyruvic transaminase 46 IU/L; and bilirubin 0.9 mg/dL.

**Figure 2:**
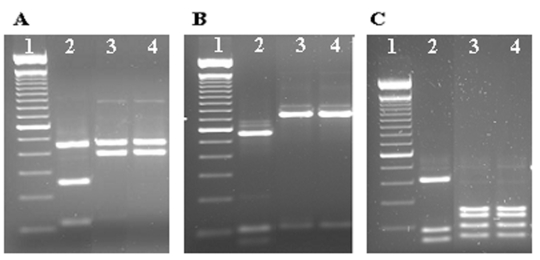
Identification of *Cryptosporidium muris* from two stool samples from a Peruvian patient using restriction fragment length polymorphism analysis of polymerase chain reaction products with *Ssp*I (A), *Vsp*I (B) and *Dde*I (C). Lane 1, 100-bp molecular markers; lane 2, *C. hominis* control; lanes 3 and 4, *C. muris* from the patient.

The diagnosis of *Cryptosporidium* in the patient’s samples was confirmed by a small subunit rRNA-based nested PCR, which amplified a portion of the rRNA gene (830 bp). *Cryptosporidium* spp. was determined by the banding patterns of restriction digestions of PCR products with *Ssp*I, *Vsp*I, and *Dde*I (*9*). Diagnosis was confirmed by DNA sequencing of three independent PCR products from each sample in both directions on an ABI PRISM 3100 (Applied Biosystems, Foster City, CA) instrument. Figure 2 shows the RFLP analysis of three PCR products from each sample with restriction enzymes *Ssp*I and *Vsp*I; these results suggest that these PCR products belonged to either *C. muris* or *Cryptosporidium andersoni* (*10*). Further RFLP analysis with *Dde*I showed banding patterns identical to *C. muris* (*9*; Figure 2). All DNA sequences obtained from the six PCR products were identical to those previously reported by Xiao et al. (*10*,*11*) from *C*. *muris* from a Bactrian camel, a rock hyrax, and mice (GenBank accession nos. AF093997 and AF093498) and another isolate recently found in an HIV patient in Kenya (*5*).

After the diagnosis of intestinal parasite infection, the patient was treated with TMP-SMX (trimethoprim 160 mg, sulfamethoxazole 800 mg) Forte twice a day for 1 week and then TMP-SMX once a day for *Pneumocystis carinii* pneumonia prophylaxis. The patient was also placed on AZT/3TC and nevirapine. The patient recovered with no further evidence of *Cyclospora*, *Blastocystis*, or *C.* *muris* in stool samples taken 2 months posttreatment. She became afebrile and had gained 5 kg as of 2 months’ posttreatment. Molecular analysis of a stool sample collected 122 days after the initial diagnosis confirmed that the patient had recovered from the *C. muris* infection.

## Conclusions

This report represents the third confirmed case of *C. muris* infection in humans. Previously, one case of *C. muris* infection was identified in an HIV-positive child in Thailand and in an HIV-positive adult in Kenya using microscopy and molecular analysis (*5*,*7*). *C. muris* and *C. andersoni*–like oocysts were found in two healthy Indonesian girls, but the diagnosis was not confirmed by molecular tools (*8*). One putative *C. muris* infection was reported in an immunocompromised patient in France based on sequence analysis of a small fragment of the SSU rRNA (*6*). However, the sequence presented was more similar to that of *C. andersoni* (2-bp differences in a 242-bp region) than to *C. muris* (8-bp differences in the region).

Although determining whether or not the *C. muris* contributed medical problems in this patient is not possible, detecting *C. muris* in her stool sample is an unexpected finding. A major difference between *C. parvum* or *C. hominis* and *C. muris,* is that *C. parvum* and *C. hominis* normally colonize the intestine, whereas *C. muris* is a gastric pathogen in cattle. Anderson (*12*) and Esteban and Anderson (*13*) reported that another gastric species, *C. andersoni,* infects only the glands of the cattle stomach (abomasum), where it retards acid production. These researchers postulated that this process may affect protein digestion in the abomasum and account for the fact that milk production in cows that are chronically infected with *C. muris* appears to be reduced by approximately 13%. Thus, an infection by *C. muris* may perhaps cause similar protein digestion problems in human infections, particularly in HIV-positive persons.

Even though only a few cases of *C. muris* infections have been identified so far in humans, gastric cryptosporidiosis occurs much more often than believed, especially in HIV-positive persons. Up to 40% of cryptosporidiosis in HIV-infected persons includes gastric involvement (*14*). Although most gastric *Cryptosporidium* infections in HIV-positive persons are likely caused by *C. parvum* or *C. hominis* because of immunosuppression, the contribution of *C. muris* probably has been underestimated. Thus, molecular characterizations of stomach tissues from patients with gastric cryptosporidiosis may help us to understand the pathogenesis of human *Cryptosporidium* infection.

Our report expands the geographic range of suspect *C. muris* infections in humans and suggests that this species may be a global emerging zoonotic pathogen. This pathogen may be of particular importance to persons living in regions where rodents live in close proximity to humans and sanitation may be minimal. *C. muris* may also be more prevalent than currently recognized. The organism is nearly twice as large as *C. parvum* and closer in size to *Cyclospora cayetanensis*. Although *Cyclospora* autofluoresces while *Cryptosporidium* does not (*15*), *C. muris* could still be easily misdiagnosed, since few laboratory workers would be familiar with *C. muris* or its morphology.

## References

[1] Fayer R, Morgan U, Upton SJ. Epidemiology of *Cryptosporidium*: transmission, detection and identification. Int J Parasitol. 2000;30:1305–22. 10.1016/S0020-7519(00)00135-1 11113257

[2] Tyzzer EE. An extracellular coccidium, *Cryptosporidium muris* (gen. et sp. nov.), of the gastric glands of the common mouse. Arch Protistenkd. 1910;26:394–418. PMC2098948 19971982

[3] Izeki M, Maekawa T, Moriya K, Uni S, Takada S. Infectivity of *Cryptosporidium muris* (strain RN 66) in various laboratory animals. Parasitol Res. 1989;75:218–22. 10.1007/BF00931279 2523540

[4] Dubey JP, Markovitis JE, Killary KA. *Cryptosporidium muris*–like infection in stomach of cynomolgus monkeys (*Macaca fascicularis*). Vet Pathol. 2002;39:363–71. 10.1354/vp.39-3-363 12014500

[5] Gatei W, Ashford RW, Beeching NJ, Kamwati SK, Greensill J, Hart CA. *Cryptosporidium muris* infection in an HIV-infected adult, Kenya. Emerg Infect Dis. 2002;8:204–6. 10.3201/eid0802.010256 11897075 PMC2732451

[6] Guyot K, Follet-Dumoulin A, Lelievre E, Sarfati C, Rabodonirina M, Nevez G, Molecular characterization of *Cryptosporidium* isolates obtained from humans in France. J Clin Microbiol. 2001;39:3472–80. 10.1128/JCM.39.10.3472-3480.2001 11574558 PMC88374

[7] Tiangtip R, Jongwutiwes S. Molecular analysis of *Cryptosporidium* species isolated from HIV-infected patients in Thailand. Trop Med Int Health. 2002;7:357–64. 10.1046/j.1365-3156.2002.00855.x 11952952

[8] Katsumata T, Hosea D, Ranuh IG, Uga S, Yanagi T, Kohno S. Short report: possible *Cryptosporidium muris* infection in humans. Am J Trop Med Hyg. 2000;62:70–2. 10761726 10.4269/ajtmh.2000.62.70

[9] Xiao L, Singh A, Limor J, Graczyk TK, Gradus S, Lal A. Molecular characterization of *Cryptosporidium* oocysts in samples of raw surface water and wastewater. Appl Environ Microbiol. 2001;67:1097–101. 10.1128/AEM.67.3.1097-1101.2001 11229897 PMC92700

[10] Xiao L, Escalante L, Yang CF, Sulaiman I, Escalante AA, Montali RJ, Phylogenetic analysis of *Cryptosporidium* parasites based on the small-subunit rRNA gene locus. Appl Environ Microbiol. 1999;65:1578–83. 10103253 10.1128/aem.65.4.1578-1583.1999 PMC91223

[11] Xiao L, Sulaiman IM, Ryan UM, Zhou L, Atwill ER, Tischler ML, Host adaptation and host-parasite co-evolution in *Cryptosporidium*: implications for taxonomy and public health. Int J Parasitol. 2002;32:1773–85. 10.1016/S0020-7519(02)00197-2 12464424

[12] Anderson BC. Cryptosporidiosis in bovine and human health. J Dairy Sci. 1998;81:3036–41. 10.3168/jds.S0022-0302(98)75868-0 9839243

[13] Esteban E, Anderson BC. *Cryptosporidium muris*: prevalence, persistency, and detrimental effect on milk production in a drylot dairy. J Dairy Sci. 1995;78:1068–72. 10.3168/jds.S0022-0302(95)76723-6 7622718

[14] Lumadue JA, Manabe YC, Moore RD, Belitsos PC, Sears CL, Clark DP. A clinicopathologic analysis of AIDS-related cryptosporidiosis. AIDS. 1998;12:2459–66. 10.1097/00002030-199818000-00015 9875584

[15] Varea M, Clavel A, Doiz O, Castillo FJ, Rubio MC, Gómez-Lus R. Fuchsin fluorescence and autofluorescence in *Cryptosporidium, Isospora* and *Cyclospora* oocysts. Int J Parasitol. 1998;28:1881–3. 10.1016/S0020-7519(98)00146-5 9925267

